# Nicotine inside neurons

**DOI:** 10.18632/oncotarget.13463

**Published:** 2016-11-18

**Authors:** Cecilia Gotti, Michele Zoli

**Affiliations:** CNR, Neuroscience Institute-Milano, Biometra University of Milan, Milan, Italy

**Keywords:** nicotine, nicotinic receptors, α4β2 subtype stoichiometry, up-regulation

Tobacco smoking is a major source of preventable morbidity and mortality worldwide particularly due to lung tumours and/or cardiovascular pathologies. Continued tobacco smoking is primarily driven by dependence on nicotine, one of the main psychoactive ingredients of tobacco smoke, which initiates a series of adaptive changes in brain neurons and circuits by binding with high affinity to neuronal nicotinic acetylcholine receptors (nAChRs) [1].

Nicotine is a highly lipophilic compound and, once in the blood stream, rapidly crosses the blood brain barrier and accumulates in the brain. nAChRs are a family of pentameric ion channels, whose variety is mainly due to the diversity of the possible homomeric or heteromeric combinations of nine α (α2−α10) and three β (β2−β4) subunits, which respond to endogenous neurotransmitter acetylcholine (ACh) and exogenous nicotine. Interestingly, the half-life of nicotine in brain tissue, is much longer than that of ACh because acetylcholinesterase does not hydrolyse nicotine, which is only metabolised by liver enzyme [2]. nAChRs are widely and unevenly distributed in the central nervous system (CNS). The majority of them have a presynaptic and/or preterminal localisation, where they modulate the release of many neurotransmitters, whereas those in the peripheral nervous system have a postsynaptic localisation and mediate fast synaptic transmission.

Chronic exposure to cigarette smoke or nicotine gives rise to neural adaptations. One prominent neuroadaptation in the brain of humans and animals chronically exposed to nicotine [3] is the cell and region-specific up-regulation of nAChRs [4] that leads to change in downstream plasticity at molecular, cellular and circuit levels [1].

The α4β2 nAChR subtype is the most highly expressed subtype in the CNS and when is expressed in heterologous systems can exist in alternate (α4β2)_2_α4 and (α4β2)_2_β2 stoichiometries that have different calcium permeability and agonist or antagonist sensitivity [5]. In both stoichiometries two ACh binding site located at α4/β2 subunit interfaces are present, but in the (α4β2)_2_α4 stoichiometry an additional ACh binding site, with low affinity for ACh, is also present at the α4/α4 interface [5].

Prolonged exposure to nicotine *in vivo*, increases the number of α4β2 nAChR binding sites as a result of an increase in α4 and β2 subunit protein levels without any change in mRNA levels (reviewed in [6]). This up-regulation in the brain is not uniform and so we analyzed the effect of chronic (14 days) nicotine on the expression of α4β2 nAChRs in the cortex and thalamus, two brain regions of C57Bl/6 mice that respond differently to nicotine [7]. We found that the α4 and β2 subunits under control conditions, are only present in assembled α4β2 receptors in the cortex and thalamus and in this latter region a significantly higher proportion of receptors have the (α4β2)_2_β2 stoichiometry.

The chronic *in vivo* administration of nicotine at a concentration that is in the range of that found in the blood of heavy smokers, more markedly up-regulated α4β2 nAChRs in the cortex than those in the thalamus. This up-regulation was paralleled by an increased proportion of receptors with (α4β2)_2_β2 stoichiometry in the cortex but not in the thalamus. The up-regulation and altered stoichiometry are transient because they returned to control levels with an average half-time of 2.7 days after nicotine withdrawal. This change in stoichiometry is very important because it can greatly influence the physiological response of neurons to nicotine and ACh.

## What may be the mechanism(s) by which nicotine interferes with nAChR expression?

The assembly of nAChRs is a slow and inefficient process. nAChR subunits cannot leave the endoplasmic reticulum (ER) until they have reached their correctly folded conformation, and any misfolded or unassembled proteins are retro-translocated from the ER to the cytoplasm for proteasomal degradation. After subunit assembly into pentamers in the ER, transport-competent nAChRs are recruited at ER exit sites and pass through COPII vesicles to the Golgi apparatus and then to the plasma membrane [2, 6]. This tight quality control guarantees that only corrected fully assembled receptors reach the cell surface. The assembly of nAChRs in the ER and their transport to the cell surface is therefore crucially important for regulating the efficacy of synaptic transmission.

Although our study does not indicate the pathways by means of which nicotine favors the increase in cortical (α4β2)_2_β2 receptors, previous studies of heterologous systems have shown that its up-regulatory effect is due to its interactions with intracellular and not surface exposed receptors [1, 2, 6]. The suggested mechanisms include: nicotine ability to assist the assembly and stabilization of pentamers (those with the (α4β2)_2_β2 stoichiometry), and to increase receptor export from the ER.

Another possible mechanism by means of which nicotine can increase the expression of nAChRs with a (α4β2)_2_β2 stoichiometry is by regulating the interaction between nAChR subunits and modulating proteins. Nichols et al., 2014, [8] have recently shown that Lynx1, a glycosylphosphatidylinositol-anchored neurotoxin-like receptor binding protein, by binding the α4 subunit in the ER favors the formation of the α4/α4 dimer and biases the formation of nAChR with the (α4β2)_2_α4 stoichiometry.

It is thus possible that the brain region-specific up-regulation of nAChRs caused by chronic nicotine exposure may at least partially be due to the differential expression of modulating proteins, such as Lynx1, in different neuronal cell types. In regions such as the cortex, where receptors with the (α4β2)_2_α4 stoichiometry prevail under control conditions, the presence of nicotine (which is known to bind to the α4/β2 interface), may hamper the physiological formation of the α4α4dimer, thus leading to the higher expression of nAChRs with the (α4β2)_2_β2 stoichiometry, and altering the intracellular receptor population that reaches the plasma membrane.

In conclusion, the crucial effects of chronic nicotine exposure are intracellular and include a cell-specific influence on the assembly and stoichiometry of the nAChRs exported to the plasma membrane, that affects the function of surface nAChRs and cholinergic transmission.

We believe that the mechanism of nicotine action on α4β2 receptors reported here may be also relevant for other nAChR subtypes. It is, indeed, worthwhile to consider extraneuronal tissues, especially where nicotine may reach much higher concentrations such as the lung epithelia. Many peripheral cell types and tissues express nAChR subunit transcripts, that may play crucial roles in signal transduction underlying tumor initiation or growth [9].

## References

[1] Pistillo F (2015). Prog Neurobiol.

[2] Henderson BJ, Lester HA (2015). Neuropharmacoly.

[3] McClure-Begley T D (2016). eNeuro.

[4] Marks M J (2011). J Pharmacol Exp Ther.

[5] Mazzaferro S (2011). J Biol Chem.

[6] Colombo S F (2013). Biochem Pharmacol.

[7] Fasoli F (2016). Neuropharmacoly.

[8] Nichols W A (2014). J Biol Chem.

[9] Grando SA (2014). Nat Rev Cancer.

